# First detection and tracing of grapevine red blotch virus (GRBV) in Australia using tiled amplicon sequencing

**DOI:** 10.1007/s00705-025-06366-7

**Published:** 2025-07-10

**Authors:** Tonny Kinene, Andrew S. Taylor, Richard Fennessy, Brenda A. Coutts, David Lovelock, Cuiping Wang, Kamalpreet Kaur, Thao Tran, Brendan Rodoni, Fiona E. Constable, Monica A. Kehoe

**Affiliations:** 1 Department of Primary Industries and Regional Development, Diagnostics and Laboratory Services, 3-Baron-Hay Court, South Perth, WA 6151 Australia; 2 Department of Primary Industries and Regional Development, 1 Verschuer Pl, Davenport, Western Australia; 3 Plant Health Australia, Level 1, 1 Phipps Close, Deakin, ACT 2600 Australia; 4 School of Applied Systems Biology, La Trobe University, Bundoora, Victoria Australia; 5 Agriculture Victoria Research, Department of Energy, Environment and Climate Action, Melbourne, Victoria Australia

## Abstract

Grapevine red blotch virus (GRBV; species *Grablovirus vitis*, genus *Grablovirus*, family *Geminiviridae*) poses a significant threat to viticulture worldwide, impacting grapevine health and wine quality. Here, we report the first detection and tracing of GRBV in Australia and provide a summary of a subsequent survey to determine the extent of GRBV distribution in Western Australia. Additionally, the study introduces a tiled amplicon sequencing method, which, when combined with long-read nanopore sequencing, enables rapid GRBV genome sequencing. Our analysis suggests a single introduction of GRBV from North America to Australia through the state of Victoria and subsequently to Western Australia. Finally, this study provides insight into the epidemiology of GRBV, based on strain variation and distribution, which is a crucial step in supporting an emergency biosecurity response and implementing effective control measures to safeguard the sustainability of the wine industry in Australia.

**Supplementary Information:**

The online version contains supplementary material available at 10.1007/s00705-025-06366-7.

## Introduction

Grapevine red blotch disease (GRBD) was first described in California in 2008 when symptoms of diseased red cultivars of *Vitis vinifera* presented in a similar manner to typical leafroll disease despite testing negative for the most common viruses. Around the same time, in 2010, similar symptoms were described on *V. vinifera* ‘Cabernet Franc’ collected from a GRBD-affected vineyard in New York and named "grapevine Cabernet Franc-associated virus" (GCFaV) [1, 2]. Finally, a virus called "grapevine red leaf-associated virus" (GRLaV) was identified in grapevines with GRBD symptoms in commercial plantings of *V. vinifera* cultivars Merlot and Cabernet Franc in Washington State [3]. It was subsequently shown that the symptoms observed in all three studies had the same causal agent, a single-stranded circular DNA virus called "grapevine red blotch virus" (GRBV; species *Grablovirus vitis*, genus *Grablovirus*, family *Geminiviridae*) [4, 5].

The genome of GRBV is 3.2 kb in size and contains eight open reading frames (ORFs), with five ORFs in the virion sense (V0, V0_2_, V1, V2, V3) and three in the complementary sense (C1, C2, C3), and these overlap each other [3, 4, 6, 7]. The GRBV genome also consists of a nonanucleotide motif associated with replication, which has been observed in most members of the family *Geminiviridae* [2]. Much is still unknown about GRBV; however, sequencing of its genome led to the development of a series of diagnostic polymerase chain reaction (PCR) assays targeting ORFs encoding the viral protein V2 [8] or the proteins V1 and C1 [2]. Phylogenetic analysis based on individual ORFs and whole genome sequences revealed two distinct clades of GRBV, with the majority of the variants belonging to clade 2 sharing about 98% nucleotide sequence identity with each other and the variants in clade 1 sharing about 95% nucleotide sequence identity with each other [2].

Since its initial discovery in the USA, GRBV has been reported in other grape-growing regions worldwide, leading in some cases to significant economic losses in the wine industry, estimated at USD$68,548 per hectare [9]. Affected locations include Canada [10], the USA [2, 8, 11], Mexico [12], Argentina [13], India [14], and Korea [15]. GRBV has also been identified in non-commercial collections in Switzerland [16], France [17], and Italy [18]. While these detections are relatively recent, GRBV has been found in an archival *Vitis* herbarium specimen collected in 1940 in California [11], suggesting that the virus had been present in the USA for many years before its first detection in 2008 in California [19]. GRBV is graft-transmissible, and its primary mode of spread is via the movement of infected material for propagation activities [8, 20]. The main insect vector associated with GRBV transmission in North America is the three-cornered alfalfa hopper (*Spissistilus festinus*) [21, 22]; however, other potential insect vectors are being investigated, including unknown Aphididae; *Aceratagallia* spp., *Acinopterus angulatus*, *Caladonus coquilleti*, and *Colladonus montanus reductus* of the family Cicadellidae; *Empoasca* spp., *Erythroneura elegantula*, *Scaphytopius graneticus*, and *Osbornellus borealis* of the family Cicadellidae, and *Stictocephala basalis* and *Stictocephala bisonia* of the family Membracidae. [22–25]. *Spissistilus festinus* is not known to occur in Australia, nor are any of the other suspected vectors.

Symptoms of GRBD include red veins, red blotches, total leaf reddening, and grapevine decline, leading to yield loss and reduced fruit and wine quality [8, 19]. However, the severity of these symptoms and their impact can vary depending on the grapevine cultivar, vineyard location, and growing season [2, 4, 19, 20]. In addition to leafroll symptoms, GRBD symptoms can also be confused with the effects of abiotic and biotic factors such as poor root health, physical injuries, nutrient deficiencies such as magnesium or potassium deficiency, or mite damage [11, 19]. These factors all combine to make diagnosis of GRBV based on symptoms challenging and unreliable. Therefore, molecular laboratory assays, such as polymerase chain reaction (PCR), isothermal amplification, and partial- or whole-genome sequencing are recommended for accurate diagnosis of GRBV [2, 20, 26].

The Western Australian grapevine germplasm collection, located at the Manjimup Horticulture Research Institute (MHRI) of the Department of Primary Industries and Regional Development (DPIRD), consists of 127 varieties suitable for table, dry, or wine grape production and 26 rootstock varieties. These varieties have been sourced over time from national and international collections. MHRI also hosts an alternative wine grape variety evaluation block and a block of Chardonnay dedicated to research purposes. A second location at the Western Australian College of Agriculture in Wokalup houses a source block with 22 grape varieties, all established from the MHRI Germplasm Collection and the alternative wine grape variety evaluation block in Manjimup. As part of an ongoing three-year general grapevine virus surveillance and monitoring program, every grapevine was tested for grapevine virus A (GVA; species *Vitivirus alphavitis*; genus *Vitivirus*, family *Betaflexiviridae*), grapevine leafroll-associated virus 1 (GLRaV1; species *Ampelovirus univitis*; genus *Ampelovirus*, family *Closteroviridae*), and grapevine leafroll-associated virus 3 (GLRaV3; species *Ampelovirus trivitis*). In 2022, the testing regime was expanded to include a broader range of viruses, including GRBV.

Here, we report the first detection of GRBV in Australia. The virus was found in the MHRI germplasm collection and the Wokalup source blocks in Western Australia in 2022, and a survey was conducted to determine the extent of its distribution. We also report the development of a tiled amplicon sequencing method, which, when combined with long-read nanopore sequencing, enables rapid GRBV genome sequencing. This method facilitated the tracing of the source of the introduction into Western Australia.

## Materials and methods

### Sample collection

In 2022, a total of 2128 grapevines were sampled across the MHRI germplasm collections, the experimental Chardonnay vineyard at MHRI, and the source blocks at Wokalup, Western Australia (Table 1). MHRI hosts the Western Australian germplasm collection of 127 alternative varieties (Supplementary Table S1) and a block of Chardonnay used for research purposes. Wokalup hosts a subset of 22 varieties from the Western Australia Germplasm collection (Supplementary Table S2). The original source of one GRBV-positive variety was traced to the Commonwealth Scientific and Industrial Research Organisation (CSIRO) germplasm collection at Irymple, Victoria, and three grapevines from each of the two clones of that variety were sampled. Grapevine samples were collected as canes, approximately 10–20 cm long, and three canes were collected per grapevine. Each sample was placed in a separate zip-lock plastic bag and labelled with grapevine, panel, and row numbers and kept at 4°C until being sent by overnight courier for testing by the diagnostic laboratories. Western Australian samples were sent to the Department of Primary Industries and Regional Development Diagnostics and Laboratory Services, and the Victorian samples were sent to Crop Health Services, Agriculture Victoria.

**Table 1:** Summary of survey sample results for testing of grapevine red blotch virus by conventional PCR, real-time PCR (qPCR), and tiled amplicon genome sequencing (TAS) methods

	PCR ([2] & [8])^1^	qPCR [29]	TAS (this study)^2^
Detected– number of individual grapevines	12	6	19
Detected– number of grapevines tested in pool	16 (2 pools)^3^	16 (2 pools)^3^	16 (2 pools)^3^
Indeterminate– number of individual grapevines	5	17	n/a
Indeterminate– number of grapevines tested in pools	10	87	n/a
Not detected– number of individual grapevines	405	369	67
Not detected– number of grapevines tested in pools	1680	1472	n/a
Total number of grapevines tested by the method either in pools and/or individually	2128	1967	102

^1^ Indicates that a grapevine was detected by one or both conventional PCR assays

^2^ TAS was performed only on individual grapevines from samples that gave a detected or indeterminate result by any one of the other methods, either individually or in a pool. Not all samples were tested using TAS. It was used to resolve discrepancies raised between the results of the conventional PCR or qPCR. Hence, no indeterminate results were obtained by this method

^3^ Represents two composite tests of eight grapevines each that were positive, with total grapevines tested equal to 16. It is therefore possible that as few as one grapevine per pool was infected with GRBV

### DNA extraction

A sterile blade was used to peel off the epidermis and bark tissue of a 10- to 20-cm-long grapevine cane, and the cambium layer was scraped with the blade at three different locations of the cane. For Western Australian samples, 100 mg (fresh weight) of the resulting tissue were placed into a 5-ml screw cap with four ball bearings. Two ml of Mackenzie buffer [27] was added to the sample, which was allowed to stand at room temperature for 1 hour. Samples were then placed in a TissueLyser (Retsch MM 400, Germany) for 2 minutes, and the DNA was extracted by following the manufacturer’s instructions in the DNeasy Plant Mini Kit protocol (QIAGEN, Hilden Germany) with the final elution volume adjusted to 50 µl. For Victorian samples, 300 mg (fresh weight) of fresh tissue was placed into a universal mesh extraction bag (Bioreba AG) and homogenised in a modified lysis buffer at a ratio of 1:10 w/v [28]. A DNeasy Plant Mini Kit (QIAGEN) was used for extraction, and DNA was eluted in 200 µl of buffer AE. Each extract was stored at -20°C until further use.

### PCR and qPCR analysis

All Western Australian and Victorian samples were screened for GRBV using a conventional PCR assay with the primer pair CPfor/Cprev [2]. The Western Australian samples were also screened using a conventional PCR assay with the primer pair GVGF1/GVGR1 [8]. To assist in resolving discrepancies between the two assays, a real-time PCR (qPCR) assay with the primer pair GRBV-F/GRBV-R [29] was also used to screen 1967 of the Western Australian samples and all Victorian samples. A small subset (approx. 10 vines) were tested using Agdia AmplifyRP Acceler8 for GRBV (Agdia Inc., USA), as an additional confirmation of the initial positive detection. The PCR and qPCR conditions for these assays were followed as described in the respective references.

Initially, all grapevines were tested in pools of up to 10 grapevines per sample, where 1680 grapevines returned negative results in those pools by both conventional PCR and qPCR (Table 1). If GRBV was not detected, the grapevines in the pooled samples were not tested any further. Pools that returned results of GRBV detected or indeterminate by either PCR or qPCR were retested as individual grapevines, and this led to 422 grapevines being retested individually. The exception was two pools of eight grapevines each in which GRBV was detected by PCR and qPCR, which were not retested individually.

Conventional PCR results were classified as detected (positive) when a clear, distinct band of the expected size was observed on the gel following electrophoresis. Results were deemed indeterminate if the band was faint, unclear, or of an incorrect size. A result was considered negative if no visible band was present on the gel. For quantitative PCR (qPCR), results were considered positive when replicate tests produced a Ct value of 30 or less and the melting temperature (Tm) was within 1 degree of the included positive control. A result was interpreted as indeterminate when Ct values between 30 and 40 were obtained or only one of the two replicates produced a Ct value or a Tm value. The test result was considered negative if no Ct or Tm value was obtained in either replicate for the sample. These values were set by each lab based on validation with known positive and negative samples to allow for differences in instruments and master mixes. All PCR-, qPCR-, or AmplifyRP Acceler8-positive or indeterminate samples from Western Australia and one sample from Victoria were then screened using the newly developed tiled amplicon sequencing assay for whole-genome recovery.

### The tiled amplicon sequencing (TAS) assay

The TAS assay was designed to cover the whole genome of GRBV, and isolate BVSE62 (GenBank accession MF795150) was used as the reference genome. The web-based primer design tool Primal Scheme (https://primalscheme.com (accessed on 16 July 2022) [30] was used to design multiplex primer sets for the amplification of the GRBV genome, with overlaps of about 20 bp and an amplicon length of 400 bp. In total, 13 pairs of primers were designed (Table 2). The primer pairs were divided into two primer pools, i.e., primer pool A, containing seven primer pairs, and primer pool B, containing six primer pairs, and a working stock of 10 µM was made for each primer pool. In a clean PCR biosafety cabinet, two individual 25-µl PCRs, one for each primer pool, were set up per sample. Both PCRs contained 12.5 µl of Q5 Hot Start High-Fidelity 2X Master Mix (New England Biolabs), 3.7 µl of one primer pool, 3.8 µl of nuclease-free water, and 5 µl of template DNA. A negative control was included in each PCR run. The cycling conditions were initial denaturation at 98°C for 30 seconds, followed by 35 cycles of denaturation at 98°C for 15 seconds and annealing and extension at 65°C for 5 minutes. The resulting PCR products from each reaction were combined in a final volume of 50 µl and purified using AMPure XP Beads (Beckman Coulter). The purified PCR product was then quantified using a Qubit 2.0 fluorometer (Qubit, Thermo Fisher Scientific) and subjected to nanopore sequencing using a Rapid Barcoding Sequencing Kit (SQK-RBK004). The sequencing was carried out on a MinION MK1C Sequencing Device using MinION flow cells (FLO-MIN106D, R9.4.1) according to the manufacturer’s instructions.

**Table 2:** Overlapping primer pairs spanning the whole genome of grapevine red blotch virus (GRBV) that were used for tiled amplicon whole-genome sequencing

Name	Sequence	Primer pool	Primer length
GRBaV_1_LEFT	ATGGTACGTGGTATTCTTGCGG	A	22
GRBaV_1_RIGHT	TAAAGCCTCATCCCCTAACCCA	A	22
GRBaV_2_LEFT	GCTGTTGTGCTAATTTCTCTCTCA	B	24
GRBaV_2_RIGHT	GCTGTCGCAATAAAACAGTCTGT	B	23
GRBaV_3_LEFT	TCGTCGTCACAGAAACTGTTAGT	A	23
GRBaV_3_RIGHT	TCCCATGGAATGCAAACTGACA	A	22
GRBaV_4_LEFT	TGTTTATTTACTTACAAGTGCGAGGA	B	26
GRBaV_4_RIGHT	TGAATATATCAGATACCGAAGGATCAGT	B	28
GRBaV_5_LEFT	ATTGCTTTAGGTACTGGTGCGG	A	22
GRBaV_5_RIGHT	TCATTACGTCCTCCACCAGACA	A	22
GRBaV_6_LEFT	TGACGAGGAATCGTTTGAATCGT	B	23
GRBaV_6_RIGHT	CATCTTCATCATCCCCAACAATGG	B	24
GRBaV_7_LEFT	GCAAGTGGACATACGTTTAGATTGT	A	25
GRBaV_7_RIGHT	ACTGTACAACGACTGTCTGGAC	A	22
GRBaV_8_LEFT	ACTGAACCTGATCGTAGTAGAACTG	B	25
GRBaV_8_RIGHT	TCCAGATAACTCTTACTATGATGCAATC	B	28
GRBaV_9_LEFT	TATGAAAGAACCCAGCCGCG	A	20
GRBaV_9_RIGHT	ATCGAAGACGGGTAAAACGCAA	A	22
GRBaV_10_LEFT	TCCGCTTTGGACGATATTTTTCAT	B	24
GRBaV_10_RIGHT	GCAAAAGCTCAGCACAACGAAA	B	22
GRBaV_11_LEFT	AATCACCAGGCTCTTCCTCCTT	A	22
GRBaV_11_RIGHT	TGGAGAATTCCTACGAGTTGCA	A	22
GRBaV_12_LEFT	AGAGATGAATTTCTGGTGGAAGGT	B	24
GRBaV_12_RIGHT	CTACACGCCTTGCTCATCTTCA	B	22
GRBaV_13_LEFT	TGAGAGTTGGCTTTGTACCGTG	A	22
GRBaV_13_RIGHT	TCAACCACTATATAAAGAGTACCAACCC	A	28

### Bioinformatics

For the Western Australian samples, the electric signal data generated by the nanopore device (MinION MK1C) was base-called, demultiplexed, and trimmed using Guppy version 6.2.11. The raw reads from each barcode were then mapped to GRBV reference MF795150 using minimap2 [31], and this resulted in a draft consensus genome sequence, which was used to assess the percentage of the genome covered by mapping and the average coverage. For the Victorian sample, Minimap2 (version 2.28) was used to map reads against the GRBV reference genome from GenBank (NC_022002). The resulting reads were then processed using the default settings of SAMtools (version 1.19.2) and Racon (version 1.4.3) for sorting, alignment, and polishing. Finally, Medaka consensus (version 1.11.3) was used to generate a consensus sequence to include in further downstream analysis. The draft consensus, which covered at least 70% of the GRBV genome, was carried forward for further analysis.

### InterARTIC pipeline

The whole-genome signal data generated by the MinION device were also analysed using the InterARTIC wrapper [32], which executes command-line workflows from the ARTIC network bioinformatic pipelines [33, 34]. The demultiplexed reads were supplied to the wrapper while maintaining the data structure from the MinION device, and a custom GRBV primer scheme available at https://github.com/Kinene1/Grape-Tile-Amplicon-Scheme/tree/main/primer-scheme/Grape/GRBV/V1 was supplied for the analysis. The custom primer scheme consisted of the following files: (1) GRBV.genes.bed, which lists the position of the genes in the GRBV virus genome, (2) GRBV.scheme.bed, which lists the primers and primer positions in the primer scheme, and (3) GRBV.reference.fasta, which is a reference genome sequence obtained from GenBank (accession no. MF795150). All of the above files were supplied to the InterARTIC pipeline together with the path to the data files from the MinION device and the path to a.csv file that matched sample names to the sample barcodes as described previously [36]. InterARTIC generates a medaka consensus genome output and a Nanopolish consensus genome output [35, 36]. The consensus genome sequences from the medaka and the Nanopolish pipelines were aligned together, and the resulting consensus sequence was used as a draft GRBV genome sequence, and when compared, this was consistent with the consensus sequence obtained by mapping raw reads using Minimap2. The resulting genome sequence was used for phylogenetic analysis where at least 70% of the genome sequence was recovered.

### Phylogenetic analysis

The 139 GRBV genome sequences obtained from GenBank and the genome sequences determined in this study were aligned using MAFFT version 7.45 [37, 38] in Geneious prime software (Biomatters). The genomes were filtered according to length and sampling metadata, and the resulting 87 sequences were used for phylogenetic analysis, which was carried out using bioinformatics tools from Nextstrain [39, 40] on a local Linux computer running ubuntu 18.04.4 LTS (Intel core i9-10900X CPU @ 3.70 GHz x 20) and Bayesian inference was run on a Setonix Supercomputer at Pawsey Supercomputing Research Centre (Perth, Western Australia). The phylogeny was inferred using Nextstrain bioinformatics tools, based on a multiple sequence alignment of GRBV genome sequences obtained from the GenBank database and the new sequences from this study. The Augur bioinformatics tool from Nextstrain was used to generate a time-resolved phylogenetic tree, which was visualised using the Nextstrain Auspice bioinformatics tool and annotated to reconstruct ancestral traits and identify mutations. The phylogenetic tree inferred by Bayesian inference was constructed using MrBayes v 3.2.7 software [41] and the GTR + I + G nucleotide substitution model. Four Markov chains were run for 5 million generations, trees were sampled every 1000 generations, and 1250 suboptimal trees were discarded at the beginning of the Markov chain Monte Carlo (MCMC) run.

### Classification of GRBV detection status

A grapevine was classified as GRBV-infected (detected) if it tested positive by one or more of the GRBV-specific assays and if more than 70% of the viral genome sequence was obtained using the TAS assay. A grapevine was classified as GRBV negative (not detected) if none of the GRBV-specific assays indicated the presence of the virus in the samples provided. Lastly, a grapevine was classified as indeterminate if no conclusive result was obtained from the GRBV-specific assays. All indeterminate samples were subsequently tested using the TAS assay, which ultimately resolved the grapevine status to either detected or not detected.

## Results

In total, 2128 Western Australian grapevines were tested for GRBV using conventional PCR assays, with 1967 tested by qPCR [29] (Table 1). Initially, all grapevines were tested in pools, with 1680 grapevines yielding negative results. Grapevines from pools with detected or indeterminate results were retested individually, resulting in 422 grapevines being retested (Table 1). Discrepancies between the methods were noted: conventional PCR detected 12 positives and five indeterminates, while the qPCR detected six positives and 17 indeterminates. Two pools of eight grapevines each gave a positive result by both methods, indicating that at least one vine in each pool contained GRBV.

**Fig. 1:**
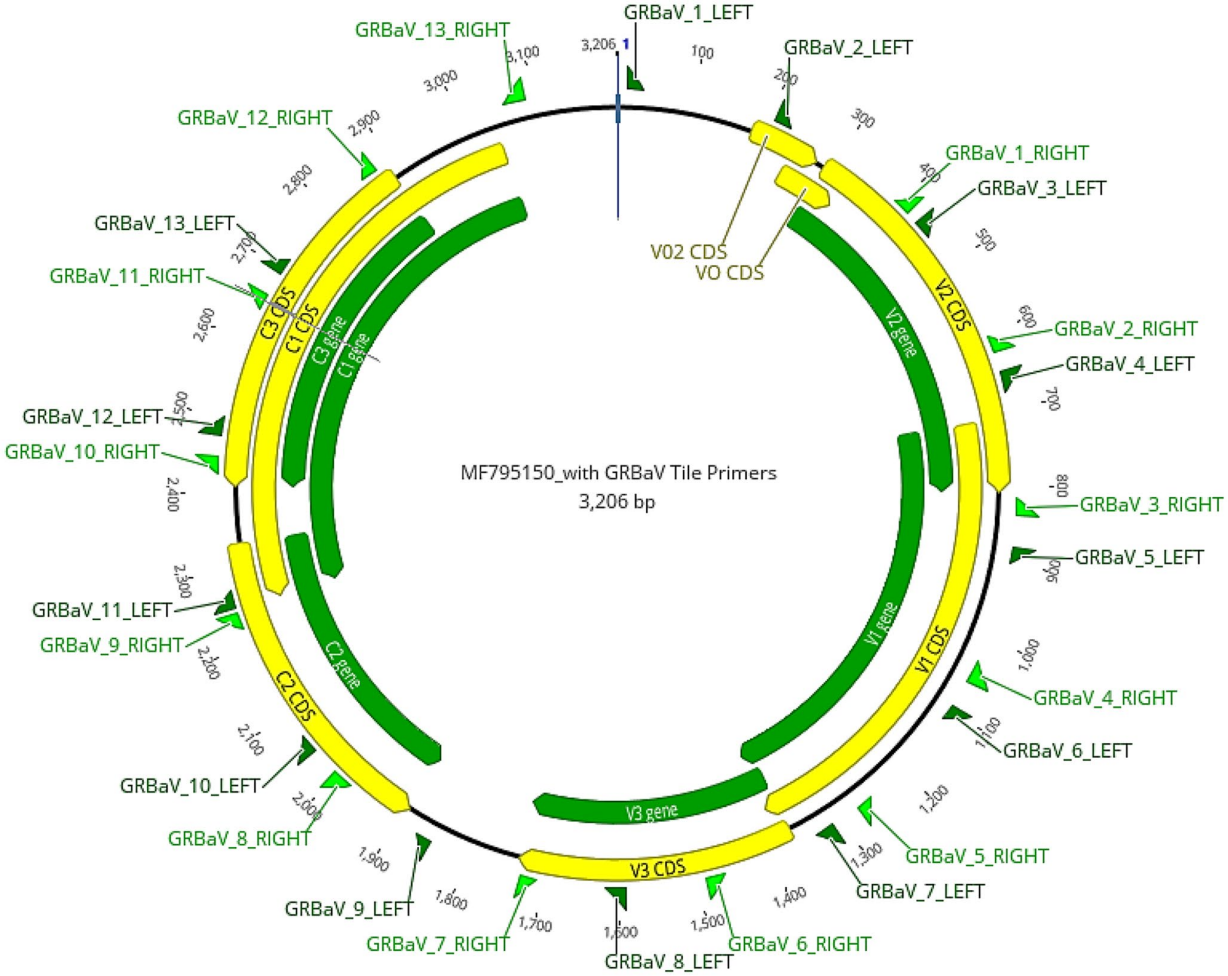
Position of primers in the grapevine red blotch virus (GRBV) genome used for targeted amplicon whole-genome sequencing

**Fig. 2:**
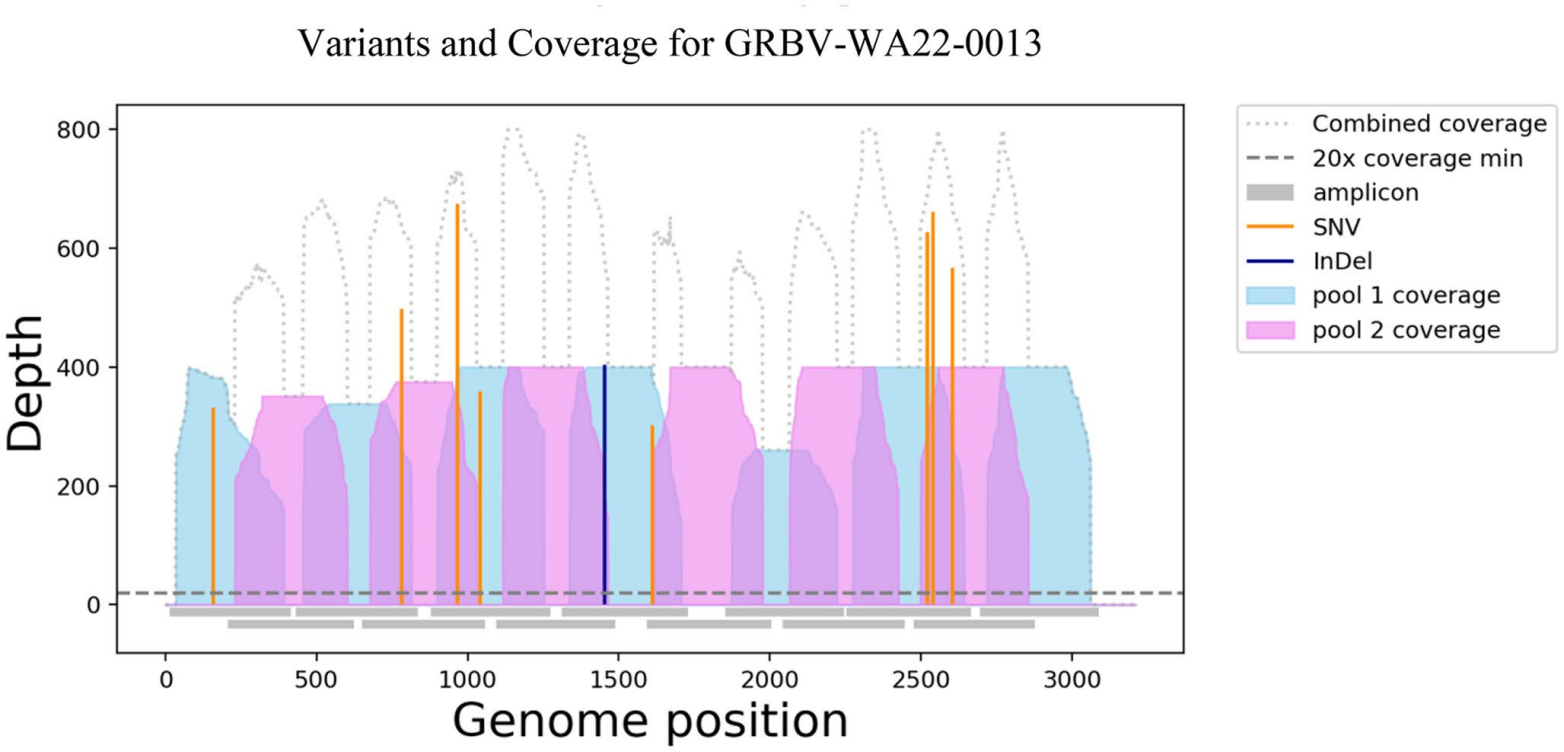
Example of the output from tile amplicon sequencing analysis of the genome of isolate GRBV-WA22-0013, using the interARTIC wrapper

A set of 13 primers pairs (Table 2) were designed for the TAS assay, and their genomic positions are indicated in Fig. 1. Using the TAS assay, 21 GRBV genome sequences with between 59.8% and 100% genome coverage were generated and confirmed the presence of the virus in 19 Western Australian grapevines, two pools of eight vines each, and in one Victorian grapevine (Table 3). For the Western Australian samples, the average read coverage across the 3.2-kb GRBV genome ranged from 865 to 11,498 reads. After mapping to the GRBV reference sequence NC_022002, the Victorian sample had 156 mapped reads with an average read coverage of 41.5. The coverage for each genome was inspected using a coverage plot generated by the interARTIC pipeline [42]. An example of this is shown for the GRBV-WA22-0013 isolate, for which all amplicons had a coverage above 20x, indicating good representation with no amplicon dropout (Fig. 2). The coverage profile plot also shows the position of single-nucleotide variations (SNVs) across the genome.

**Table 3:** Confirmation of PCR or real-time (qPCR) results by the tiled amplicon sequencing (TAS) assay and comparison with the other four assays

Sample ID	GenBank accession no.	Variety	Assay results					Whole-genome sequence data				
								Total reads	InterArtic	Map to reference MF795150		
			PCR [8]	qPCR [29]	PCR [2]	AgDia Amplify	TAS (WGS)		% Genome covered	No. of reads mapped	% Genome covered	Average coverage
GRBV-WA22-0001	OR730839	Perle de Csaba	Detected	Detected	Detected	Detected (crude)	Detected	177,086	94.26	155,176	99.75	11,498
GRBV-WA22-0002	OR730840	Malbec	Detected	Indeterminate	Detected	-	Detected	107,278	89.24	43,704	93.89	3,648
GRBV-WA22-0003	OR730841	Savagnin Blanc	Detected	Detected	-	nt	Detected	239,759	84.09	11,613	99.47	1,039
GRBV-WA22-0004	OR730842	Kadarka	Detected (composite)	Detected (composite)	nt	nt	Detected (composite)	107,709	89.11	32,767	95.54	3,370
GRBV-WA22-0005	OR730843	Kadarka	Detected (composite)	Detected (composite)	nt	nt	Detected (composite)	89,989	86.43	18,279	95.29	1,957
GRBV-WA22-0006	OR730844	Harslevelu	Detected	Indeterminate	-	nt	Detected	288104	83.94	16,074	94.23	1,452
GRBV-WA22-0007	OR730845	Harslevelu	Detected	Detected	-	nt	Detected	173,777	84	10,861	98.22	1,010
GRBV-WA22-0008	OR730846	Brachetto	Detected	Indeterminate	-	nt	*Detected	330,609	73.14	9,019	74.98	865
GRBV-WA22-0009	OR730847	Brachetto	Detected	Detected	-	nt	Detected	251,602	83.94	24,185	99.84	2198
GRBV-WA22-0010	OR730848	Brachetto	Detected	Detected	-	nt	Detected	186,482	89.49	29,218	93.33	2,711
GRBV-WA22-0011	OR730849	Brachetto	Detected	Detected	-	nt	Detected	204,049	73.71	14,388	95.13	1,646
GRBV-WA22-0012	OR730850	Chardonnay	Indeterminate	Indeterminate	nt	Detected	Detected	209,473	91.48	101,706	96.29	7,147
GRBV-WA22-0013	OR730851	Perle de Csaba	Detected	Detected	Dectected	Detected (crude)	Detected	47,343	99.91	44,794	99.72	3,653
GRBV-WA22-0014	OR730853	Harslevelu	Detected	-	-	Detected (crude)	Detected*	152,971	22.65	4,608	59.83	930
GRBV-WA22-0015	OR730854	Kadarka	-	Indeterminate	Detected	Detected (crude)	Detected*	313,830	75.76	87,795	83.22	10,521
GRBV-WA22-0016	OR730855	Brachetto	-	Indeterminate	-	nt	Detected*	163834	49.75	6,607	70.9	871
GRBV-WA22-0017	OR730856	Brachetto	Detected	Detected	-	nt	Detected*	278,201	76.89	11,967	83.34	1,288
GRBV-WA22-0018	OR730857	Merlot	-	Indeterminate	nt	nt	Detected*	71,997	63.54	3,359	85.34	360
GRBV-WA22-0019	OR730858	Merlot	Indeterminate	nt	nt	-	Detected*	219,270	75.2	24,194	83.31	2,329
GRBV-WA22-0020	OR730859	Chardonnay	Indeterminate	Indeterminate	nt	Detected	Detected*	81,284	60.36	9,030	76.01	1,039
sample3PerledeCsaba	OR730852	Perle de Csaba	nt	Detected	Detected	nt	Detected	NA	100	1156	99.3	41.5

nt, test not carried out; composite, a pool of eight grapevines tested together as one sample; crude, sample extracted using AgDia Amplify extraction buffer without purification; -, negative result

**Fig. 3:**
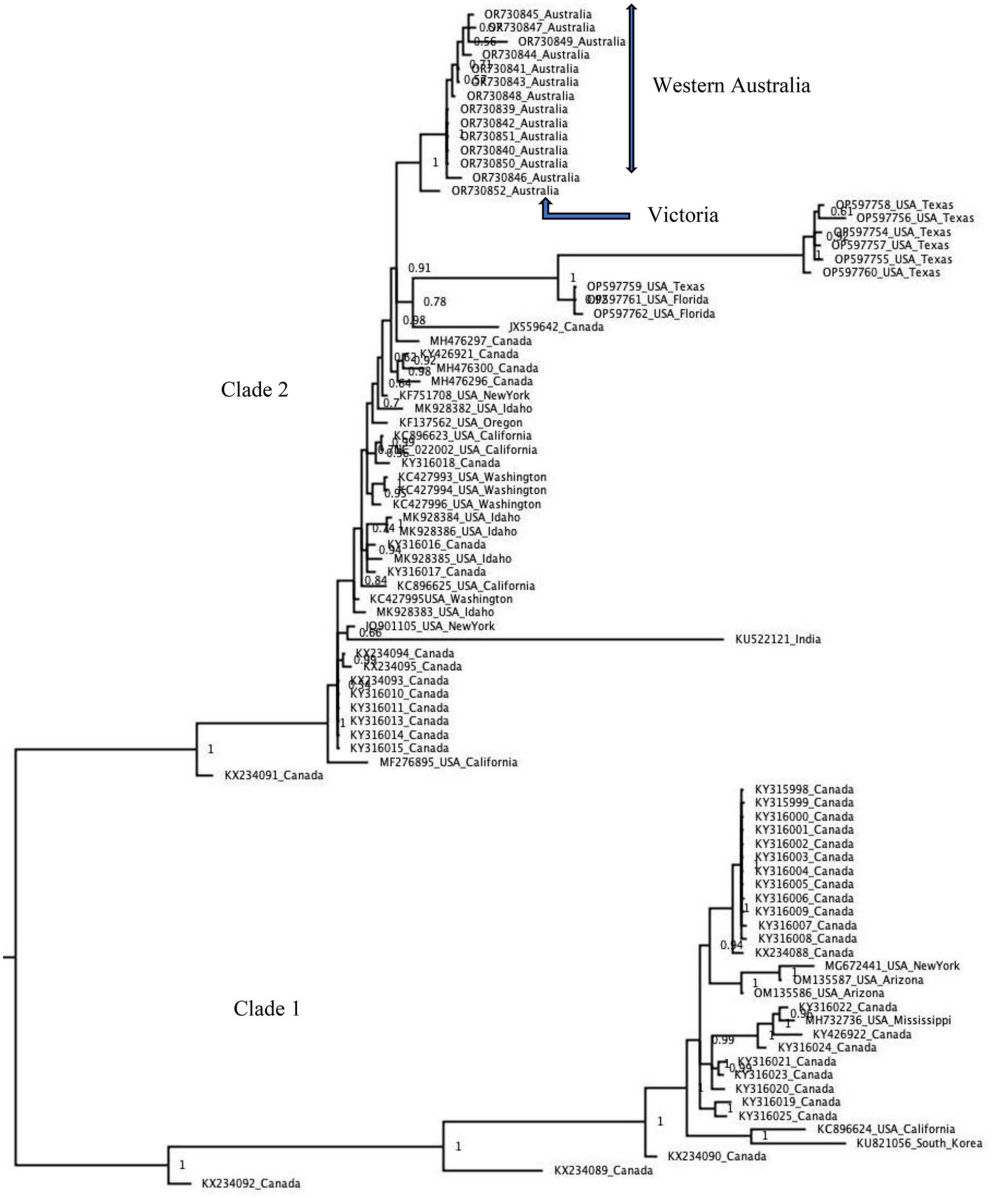
Bayesian phylogenetic tree created from an alignment of 87 grapevine red blotch virus (GRBV) genome sequences and rooted at the midpoint. The branch length is proportional to the number of nucleotide substitutions per site, and the values shown at the nodes represent the posterior probability for clade support. The Australian GRBV isolates from Western Australia and Victoria formed a separate branch in clade 2

**Fig. 4:**
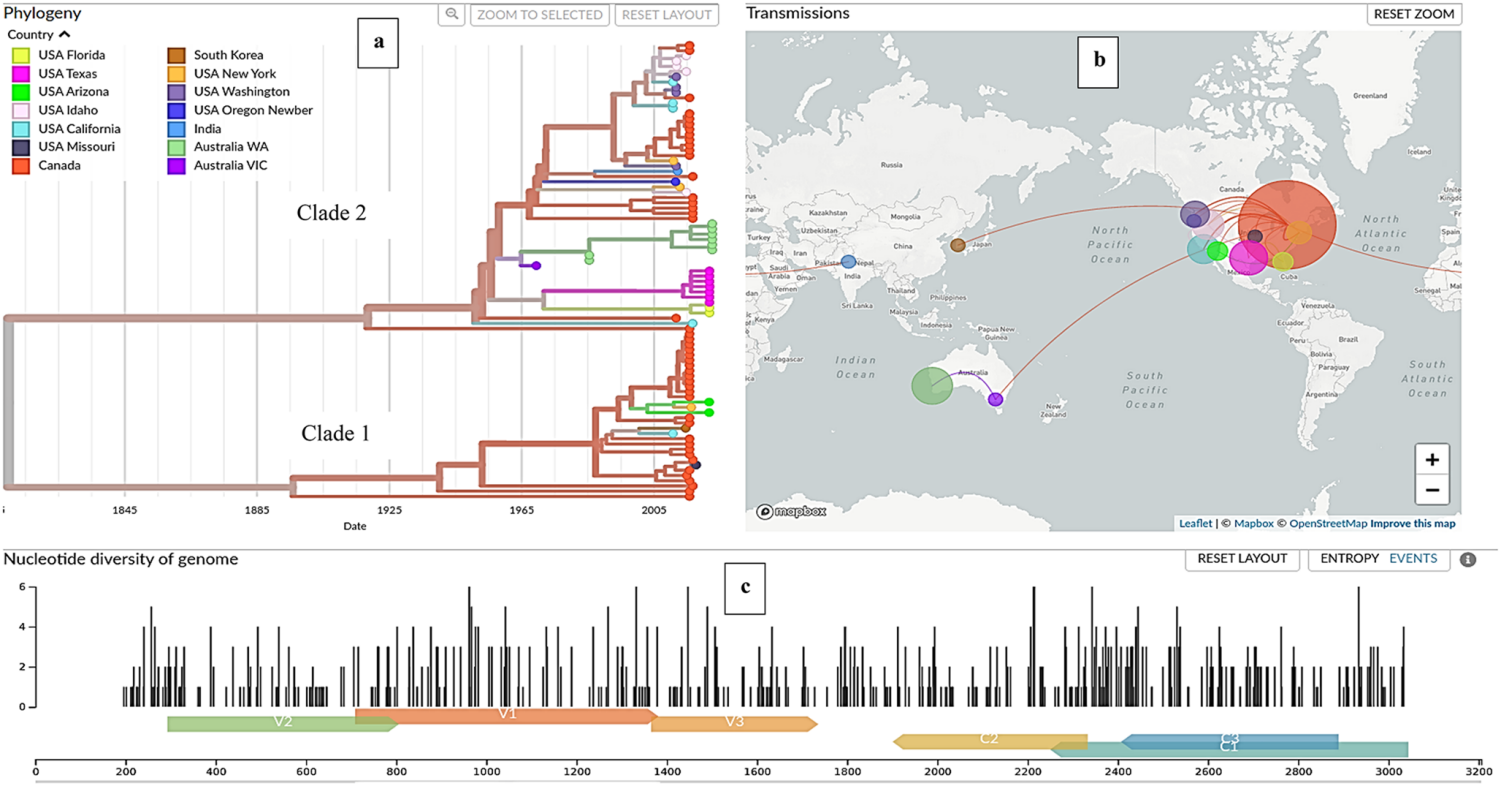
(**a**) Time-resolved phylogenetic tree based on 87 genome sequences of viruses sampled between Sept 2010 and Nov 2022. The tree branches are coloured according to the geographic location (city or country) where the isolate was collected. (**b**) Transmission map showing the spread of GRBV from North America to Australia and Asia. This map shows major transmission routes and potential global spread of GRBV. (**c**) Nucleotide diversity panel of the GRBV genome. This plot shows the degree of genetic variation across the different regions of the GRBV genome. The figures were generated using the Nextstrain Bioinformatics tool and resources

Bayesian phylogenetic analysis of 87 complete GRBV genome sequences showed that the Australian GRBV isolates clustered in clade 2 and revealed that the Victorian isolate shares a common ancestor with the Western Australia isolates (Fig. 3), with 99% nucleotide sequence identity. Canadian and USA isolates are represented in both clades, but the majority of the USA isolates were in clade 2. These results are consistent with those of the time-resolved phylogenetic tree, in which the Australian isolates were observed in clade 2 (Fig. 4a). In the time-resolved phylogenetic tree, the isolates from Western Australia and Victoria shared a common ancestor estimated to have existed around 1964, with a confidence interval ranging from 1959 to 1969. The tree shows a speciation event of the two clades at an old ancestor appearing around 1809 with an estimated confidence interval of 1685–1928. The tip dates shown on the time tree in Fig. 4a represent sampling dates apart from those of Perle de Csaba isolates in Western Australia and Victoria, which represent actual (calibration) dates of introduction, which are 1985 and 1969 respectively.

The disease transmission maps shown in Fig. 4b illustrate the geographical distribution and spread of GRBV and show the number of infected samples in a particular geographic region, represented by the area of the circle. The data indicate that GRBV moved from North America to Australia through Victoria state and then to Western Australia. A similar transmission dynamic was observed from North America to countries in Asia (South Korea and India). The diversity panel shows the number of variations accumulated across the genome of GRBV, with ORF C3 having more variation than V1 and V2 (Fig. 4c). The results of this analysis are available at https://github.com/Kinene1/Nextstrain-build-for-Grapevine-red-blotch-virus.

## Discussion

GRBV was detected in June 2022 for the first time in Australia in eight grapevine varieties, including Perle de Csaba, through routine sample screening by PCR, qPCR, and/or Amplify assays, in two germplasm collections at Manjimup and Wokalup in Western Australia. It was subsequently detected by PCR and qPCR in two clones of Perle de Csaba in the Victorian germplasm collection, which appear to be an original source of the virus in Western Australia. The presence of GRBV in Australian grapevines has been traced using the TAS assay designed for this study. This assay was combined with the portable genome sequencing technology from Oxford Nanopore Technologies to sequence GRBV genomes, enabling detailed investigation into the spread and origin of the virus. The TAS assay provides a rapid and cost-effective alternative to whole-genome sequencing using metagenomic approaches, allowing rapid characterisation and tracing of the spread of GRBV in Australia. The results of this study have important implications for the management of the disease caused by this virus and the development of effective control measures.

A comparison of our TAS assay with currently available diagnostic methods for detection of Australian GRBV isolates demonstrated its effectiveness. Rapid sequencing methods such as TAS enable us to monitor the spread of these viruses and to be quickly alerted to introductions of additional strains so that we may differentiate existing strains from new ones that may be more damaging, either on their own or in coinfections with other grapevine viruses.

Our survey showed that GRBV had not yet been detected in commercial vineyards in Western Australia, but it is important to acknowledge that the affected varieties are not common in wine or table grape production in Western Australia and that there has been only limited sampling for GRBV in commercial vineyards. It should also be noted that the insect vector responsible for secondary disease transmission in North America, known as the three-cornered alfalfa hopper (*Spissistilus festinus*) [21, 22], is not present in Australia. Therefore, how this virus might be spread by a vector in Australia is unknown. In the absence of a key vector, alternative transmission routes, such as grafting of infected plant material and root grafting should be considered [19, 20, 43]. Researchers and industry experts in Australia should evaluate potential vectors that have been identified in other countries [22, 23, 25], including unknown *Aphididae* species, the cicadellids *Aceratagallia* spp., *Acinopterus angulatus*, *Caladonus coquilleti*, *Colladonus montanus reductus*, *Empoasca* spp., *Erythroneura elegantula*, *Scaphytopius graneticus*, and *Osbornellus borealis* and the membracids *Stictocephala basalis* and *Stictocephala bisonia*.

It is clear from the existing research that visual diagnosis of GRBV infection may not always be reliable due to the similarity of foliar symptoms to those of leafroll diseases. Thus, PCR-based assays are recommended for the accurate diagnosis of GRBV [2, 8, 20]. However, in our hands, the PCR and qPCR assays used in this survey sometimes gave inconsistent results. As with many grapevine viruses, results from PCR assays can be influenced by the viral titre in the grapevine itself, since the virus is not evenly distributed. Previous studies have documented that the distribution of GRBV can fluctuate depending on the tissue type and sampling time [44]. Taking these factors into account is crucial when diagnosing and managing infections. It is also possible that a particular PCR assay may not be sensitive due to sequence variations that affect detection efficiency. The two recognised clades of GRBV differ by as much as 11%, which should be kept in mind when designing new diagnostic assays.

Bayesian phylogenetic analysis based on 87 genome sequences (Fig. 3) confirmed the two distinct clades of GRBV as well as our time-resolved phylogenetic tree (Fig. 4a), described in previous studies [2, 19]. Both phylogenetic analyses in this study showed the Australian isolates clustering together in clade 2, with the Western Australian isolates sharing a common ancestor with the Victorian isolate. The analysis from the time-resolved phylogenetic tree shows tip dates as sampling dates apart from those of Perle de Csaba isolate in Australia, which represent dates of introduction. Old records show that the white table grape variety Perle de Csaba that was found to be positive for GRBV in this study was imported into Victoria, Australia, from the University of California collection in Davis, USA, in 1969 [45] and later introduced into Western Australia in 1985. Perle de Csaba held in the National Clonal Germplasm Repository in California is now known to be infected with GRBV [46], and this USA isolate exhibits a high degree of sequence similarity (> 99% identity) to Western Australian isolates (Al Rwahnih, personal communication). Therefore, GRBV is likely to have been present in Australia for more than 53 years after introduction of infected planting material from the USA despite not being detected in Western Australia until 2022. The longstanding presence of GRBV in Australia is also implied by the results of a study by Reynard et al. [17] in which the virus was detected in two accessions in the Vassal collection in France (Kandahar 2908Mtp1 and Hussein 2904Mtp1), which were imported from Australia in 1982.

GRBV has been detected in South Korea and India, suggesting the potential spread of this virus in Asia. The map shown in Fig. 4b, which shows possible virus dispersal pathways, indicates that the virus in Asia also likely originated from the USA. This long-distance spread is primarily attributed to the unintentional movement of infected germplasm, mirroring the situation observed in Australia. Although the map suggests a directional flow (Fig. 4b), the possibility of separate introductions into Victoria and Western Australia has not been definitively ruled out. However, our phylogenetic analysis shows a high level of genetic similarity between GRBV sequences from both regions, indicating a common origin. Furthermore, historical records of plant material movement between germplasm collections provide additional support for a single-introduction scenario [45].

### Limitations of the TAS assay

The TAS assay developed in this study is not ideal for discovery of new grapevine viruses due to the specific virus primer scheme. However, it does work well for detection of GRBV through rapid genome sequencing. It is important to note that this assay involves several manual pipetting steps, which pose the risk of cross-contamination of samples. Therefore, we recommend separation of individual steps, such as extraction, reaction setup, and addition of nucleic acid to reactions, and the use of suitable negative and positive controls throughout the assay. While the assay has not been tested on the new kit chemistry (V14), it is expected to work well with the new V14 kits produced by ONT. It is worth noting that this kind of assay generates large datasets, and data storage can become a challenge. However, this can be overcome by compressing the files or changing the file format of the nanopore signal data to pod5 https://github.com/nanoporetech/pod5-file-format. Lastly, sampling bias and lack of detail in the metadata (e.g., accurate sampling dates) can obscure transmission links, such as those shown in Fig. 4b, and this may lead to a biased interpretation of the transmission dynamics data.

## Conclusion

Grapevine red blotch virus (GRBV) has been detected in Western Australia and subsequently in Victoria. These detections represent the first reports of this virus in Australia. The grapevine cultivars that tested positive were distributed in small quantities, as they are not commercially popular. The situation might have been different had the introduction and detection occurred in varieties like Shiraz or Cabernet Sauvignon. In Australia, the ability to track and detect the spread of this virus from the germplasm collection was of critical importance, thus necessitating a method for rapid genome sequencing. Currently, there is no known vector for efficient transmission of the virus in Australia, and the potential for spread via vectors remains unknown.

The affected grapevines at both locations in Western Australia have been removed from the respective collections in an effort to maintain its health status. At the time of writing, GRBV is still considered an exotic virus in Australian biosecurity. Therefore, any detections must be reported to the relevant authorities.

## Electronic Supplementary Material

Below is the link to the electronic supplementary material

Supplementary Material 1

## Data Availability

The nucleotide sequences determined in this study are available in the National Center for Biotechnology Information (NCBI) GenBank database under the accession numbers OR730839-OR730859.

## References

[1] Krenz B, Thompson JR, Fuchs M, Perry KL (2012) Complete Genome Sequence of a New Circular DNA Virus from Grapevine. J Virol 86:7715–7715. 10.1128/jvi.00943-12 22733880 10.1128/JVI.00943-12 PMC3416304

[2] Krenz B, Thompson JR, McLane HL, Fuchs M, Perry KL (2014) Grapevine red blotch-associated virus Is Widespread in the United States. Phytopathology® 104:1232–1240. 10.1094/PHYTO-02-14-0053-R 24805072 10.1094/PHYTO-02-14-0053-R

[3] Yepes LM, Cieniewicz E, Krenz B, McLane H, Thompson JR, Perry KL, Fuchs M (2018) Causative Role of Grapevine Red Blotch Virus in Red Blotch Disease. Phytopathology® 108:902–909. 10.1094/PHYTO-12-17-0419-R 29436986 10.1094/PHYTO-12-17-0419-R

[4] Cieniewicz E, Perry K, Fuchs M (2017) Grapevine Red Blotch: Molecular Biology of the Virus and Management of the Disease. In: Meng B, Martelli GP, Golino DA, Fuchs M (eds) Grapevine Viruses: Molecular Biology, Diagnostics and Management. Springer International Publishing, Cham, pp 303–314

[5] Genus Grablovirus| ICTV. https://ictv.global/report/chapter/geminiviridae/geminiviridae/grablovirus. Accessed 10 Jul 2023

[6] Vargas-Asencio J, Liou H, Perry KL, Thompson JR (2019) Evidence for the splicing of grablovirus transcripts reveals a putative novel open reading frame. J Gen Virol 100:709–720. 10.1099/jgv.0.001234 30775960 10.1099/jgv.0.001234

[7] Krenz B, Fuchs M, Thompson JR (2023) Grapevine red blotch disease: A comprehensive Q&A guide. PLOS Pathog 19:e1011671. 10.1371/journal.ppat.1011671 37824437 10.1371/journal.ppat.1011671 PMC10569545

[8] Al Rwahnih M, Dave A, Anderson MM, Rowhani A, Uyemoto JK, Sudarshana MR (2013) Association of a DNA Virus with Grapevines Affected by Red Blotch Disease in California. Phytopathology® 103:1069–1076. 10.1094/PHYTO-10-12-0253-R 23656312 10.1094/PHYTO-10-12-0253-R

[9] Ricketts KD, Gómez MI, Fuchs MF, Martinson TE, Smith RJ, Cooper ML, Moyer MM, Wise A (2017) Mitigating the Economic Impact of Grapevine Red Blotch: Optimizing Disease Management Strategies in U.S. Vineyards. Am J Enol Vitic 68:127–135. 10.5344/ajev.2016.16009

[10] Poojari S, Lowery DT, Rott M, Schmidt AM, Úrbez-Torres JR (2017) Incidence, distribution and genetic diversity of Grapevine red blotch virus in British Columbia. Can J Plant Pathol 39:201–211. 10.1080/07060661.2017.1312532

[11] Al Rwahnih M, Rowhani A, Golino D (2015) First Report of Grapevine red blotch-associated virus in Archival Grapevine Material From Sonoma County, California. Plant Dis 99:895–895. 10.1094/PDIS-12-14-1252-PDN

[12] Gasperin-Bulbarela J, Licea-Navarro AF, Pino-Villar C, Hernández-Martínez R, Carrillo-Tripp J (2019) First Report of Grapevine Red Blotch Virus in Mexico. Plant Dis 103:381–381. 10.1094/PDIS-07-18-1227-PDN

[13] Luna F, Debat H, Moyano S, Zavallo D, Asurmendi S, Gomez-Talquenca S (2019) First report of grapevine red blotch virus infecting grapevine in Argentina. J Plant Pathol 101:1239–1239. 10.1007/s42161-019-00298-3

[14] Marwal A, Kumar R, Paul Khurana SM, Gaur RK (2019) Complete nucleotide sequence of a new geminivirus isolated from Vitis vinifera in India: a symptomless host of Grapevine red blotch virus. VirusDisease 30:106–111. 10.1007/s13337-018-0477-x 31143838 10.1007/s13337-018-0477-x PMC6517466

[15] Lim S, Igori D, Zhao F, Moon JS, Cho I-S, Choi G-S (2016) First Report of Grapevine red blotch-associated virus on Grapevine in Korea. Plant Dis 100:1957–1957. 10.1094/PDIS-03-16-0283-PDN

[16] Reynard J-S, Brodard J, Dubuis N, Zufferey V, Schumpp O, Schaerer S, Gugerli P (2018) Grapevine red blotch virus: Absence in Swiss Vineyards and Analysis of Potential Detrimental Effect on Viticultural Performance. Plant Dis 102:651–655. 10.1094/PDIS-07-17-1069-RE 30673492 10.1094/PDIS-07-17-1069-RE

[17] Reynard J-S, Brodard J, Dubuis N, Kellenberger I, Spilmont A-S, Roquis D, Maliogka V, Marchal C, Dedet S, Gning O, Croll D, Gindro K, Schumpp O, Spring J-L, Lacombe T (2022) Screening of grapevine red blotch virus in two European ampelographic collections. J Plant Pathol 104:9–15. 10.1007/s42161-021-00952-9

[18] Bertazzon N, Migliaro D, Rossa A, Filippin L, Casarin S, Giust M, Brancadoro L, Crespan M, Angelini E (2021) Grapevine red blotch virus is sporadically present in a germplasm collection in Northern Italy. J Plant Dis Prot 128:1115–1119. 10.1007/s41348-021-00468-5

[19] Sudarshana MR, Perry KL, Fuchs MF (2015) Grapevine Red Blotch-Associated Virus, an Emerging Threat to the Grapevine Industry. Phytopathology® 105:1026–1032. 10.1094/PHYTO-12-14-0369-FI 25738551 10.1094/PHYTO-12-14-0369-FI

[20] Poojari S, Alabi OJ, Fofanov VY, Naidu RA (2013) A Leafhopper-Transmissible DNA Virus with Novel Evolutionary Lineage in the Family Geminiviridae Implicated in Grapevine Redleaf Disease by Next-Generation Sequencing. PLoS ONE 8:e64194. 10.1371/journal.pone.0064194 23755117 10.1371/journal.pone.0064194 PMC3673993

[21] Bahder BW, Zalom FG, Sudarshana MR (2016) An Evaluation of the Flora Adjacent to Wine Grape Vineyards for the Presence of Alternative Host Plants of Grapevine red blotch-associated virus. Plant Dis 100:1571–1574. 10.1094/PDIS-02-16-0153-RE 30686219 10.1094/PDIS-02-16-0153-RE

[22] Cieniewicz EJ, Pethybridge SJ, Loeb G, Perry K, Fuchs M (2018) Insights Into the Ecology of Grapevine red blotch virus in a Diseased Vineyard. Phytopathology® 108:94–102. 10.1094/PHYTO-07-17-0239-R 28945519 10.1094/PHYTO-07-17-0239-R

[23] Kahl D, Úrbez-Torres JR, Kits J, Hart M, Nyirfa A, Lowery DT (2021) Identification of candidate insect vectors of Grapevine red blotch virus by means of an artificial feeding diet. Can J Plant Pathol 43:905–913. 10.1080/07060661.2021.1930174

[24] LaFond HF, Volenberg DS, Schoelz JE, Finke DL (2022) Identification of Potential Grapevine Red Blotch Virus Vector in Missouri Vineyards. Am J Enol Vitic 73:247–255. 10.5344/ajev.2022.21056

[25] Wilson H, Hogg BN, Blaisdell GK, Andersen JC, Yazdani AS, Billings AC, Ooi KM, Soltani N, Almeida RPP, Cooper ML, Al Rwahnih M, Daane KM (2022) Survey of Vineyard Insects and Plants to Identify Potential Insect Vectors and Noncrop Reservoirs of Grapevine Red Blotch Virus. PhytoFrontiers^™^ 2:66–73. 10.1094/PHYTOFR-04-21-0028-R

[26] Li R, Fuchs MF, Perry KL, Mekuria T, Zhang S (2017) Development of a Fast Amplifyrp Acceler8 Diagnostic Assay for Grapevine Red Blotch Virus. J Plant Pathol 99:657–662

[27] MacKenzie DJ, McLean MA, Mukerji S, Green M (1997) Improved RNA Extraction from Woody Plants for the Detection of Viral Pathogens by Reverse Transcription-Polymerase Chain Reaction. Plant Dis 81:222–226. 10.1094/PDIS.1997.81.2.222 30870901 10.1094/PDIS.1997.81.2.222

[28] Green MJ, Thompson DA, MacKenzie DJ (1999) Easy and Efficient DNA Extraction from Woody Plants for the Detection of Phytoplasmas by Polymerase Chain Reaction. Plant Dis 83:482–485. 10.1094/PDIS.1999.83.5.482 30845543 10.1094/PDIS.1999.83.5.482

[29] Poojari S, Alabi OJ, Okubara PA, Naidu RA (2016) SYBR® Green-based real-time quantitative reverse-transcription PCR for detection and discrimination of grapevine viruses. J Virol Methods 235:112–118. 10.1016/j.jviromet.2016.05.013 27246908 10.1016/j.jviromet.2016.05.013

[30] Quick J, Grubaugh ND, Pullan ST, Claro IM, Smith AD, Gangavarapu K, Oliveira G, Robles-Sikisaka R, Rogers TF, Beutler NA, Burton DR, Lewis-Ximenez LL, de Jesus JG, Giovanetti M, Hill SC, Black A, Bedford T, Carroll MW, Nunes M, Alcantara LC, Sabino EC, Baylis SA, Faria NR, Loose M, Simpson JT, Pybus OG, Andersen KG, Loman NJ (2017) Multiplex PCR method for MinION and Illumina sequencing of Zika and other virus genomes directly from clinical samples. Nat Protoc 12:1261–1276. 10.1038/nprot.2017.066 28538739 10.1038/nprot.2017.066 PMC5902022

[31] Li H (2018) Minimap2: pairwise alignment for nucleotide sequences. Bioinformatics 34:3094–3100. 10.1093/bioinformatics/bty191 29750242 10.1093/bioinformatics/bty191 PMC6137996

[32] Ferguson JM, Gamaarachchi H, Nguyen T, Gollon A, Tong S, Aquilina-Reid C, Bowen-James R, Deveson IW (2022) InterARTIC: an interactive web application for whole-genome nanopore sequencing analysis of SARS-CoV-2 and other viruses. Bioinformatics 38:1443–1446. 10.1093/bioinformatics/btab846 34908106 10.1093/bioinformatics/btab846 PMC8826086

[33] Loman N, Rowe W, Rambaut A (2020) nCoV-2019 novel coronavirus bioinformatics protocol. In: Artic Netw. https://artic.network/ncov-2019/ncov2019-bioinformatics-sop.html. Accessed 6 May 2025

[34] Tyson JR, James P, Stoddart D, Sparks N, Wickenhagen A, Hall G, Choi JH, Lapointe H, Kamelian K, Smith AD, Prystajecky N, Goodfellow I, Wilson SJ, Harrigan R, Snutch TP, Loman NJ, Quick J (2020) Improvements to the ARTIC multiplex PCR method for SARS-CoV-2 genome sequencing using nanopore. 10.1101/2020.09.04.283077. bioRxiv 2020.09.04.283077

[35] Oxford Nanopore Technologies (2022) Medaka. https://github.com/nanoporetech/medaka. Accessed 21 Oct 2022

[36] Simpson J (2022) Nanopolish. https://github.com/jts/nanopolish. Accessed 21 Oct 2022

[37] Katoh K, Standley DM (2013) MAFFT Multiple Sequence Alignment Software Version 7: Improvements in Performance and Usability. Mol Biol Evol 30:772–780. 10.1093/molbev/mst010 23329690 10.1093/molbev/mst010 PMC3603318

[38] Katoh K, Misawa K, Kuma K, Miyata T (2002) MAFFT: a novel method for rapid multiple sequence alignment based on fast Fourier transform. Nucleic Acids Res 30:3059–3066. 10.1093/nar/gkf436 12136088 10.1093/nar/gkf436 PMC135756

[39] Hadfield J, Megill C, Bell SM, Huddleston J, Potter B, Callender C, Sagulenko P, Bedford T, Neher RA (2018) Nextstrain: real-time tracking of pathogen evolution. Bioinformatics 34:4121–4123. 10.1093/bioinformatics/bty407 29790939 10.1093/bioinformatics/bty407 PMC6247931

[40] Sagulenko P, Puller V, Neher RA (2018) TreeTime: Maximum-likelihood phylodynamic analysis. Virus Evol 4:vex042. 10.1093/ve/vex042 29340210 10.1093/ve/vex042 PMC5758920

[41] Ronquist F, Teslenko M, van der Mark P, Ayres DL, Darling A, Höhna S, Larget B, Liu L, Suchard MA, Huelsenbeck JP (2012) MrBayes 3.2: Efficient Bayesian Phylogenetic Inference and Model Choice Across a Large Model Space. Syst Biol 61:539–542. 10.1093/sysbio/sys029 22357727 10.1093/sysbio/sys029 PMC3329765

[42] Ferguson JM, Gamaarachchi H, Nguyen T, Gollon A, Tong S, Aquilina-Reid C, Bowen-James R, Deveson IW (2022) InterARTIC: an interactive web application for whole-genome nanopore sequencing analysis of SARS-CoV-2 and other viruses. Bioinformatics 38:1443–1446. 10.1093/bioinformatics/btab846 34908106 10.1093/bioinformatics/btab846 PMC8826086

[43] Flasco M, Hoyle V, Cieniewicz EJ, Fuchs M (2023) Transmission of Grapevine Red Blotch Virus: A Virologist’s Perspective of the Literature and a Few Recommendations. Am J Enol Vitic 74. 10.5344/ajev.2023.23020

[44] Setiono FJ, Chatterjee D, Fuchs M, Perry KL, Thompson JR (2018) The Distribution and Detection of Grapevine red blotch virus in its Host Depend on Time of Sampling and Tissue Type. Plant Dis 102:2187–2193. 10.1094/PDIS-03-18-0450-RE 30226420 10.1094/PDIS-03-18-0450-RE

[45] b. Dry I, Davies C, d. Dunlevy J, m. Smith H, r. Thomas M, r. Walker A, r. Walker R, r. Clingeleffer P (2022) Development of new wine-, dried- and tablegrape scions and rootstocks for Australian viticulture: past, present and future. Aust J Grape Wine Res 28:177–195. 10.1111/ajgw.12552

[46] Al Rwahnih M, Rowhani A, Golino DA, Islas CM, Preece JE, Sudarshana MR (2015) Detection and genetic diversity of Grapevine red blotch-associated virus isolates in table grape accessions in the National Clonal Germplasm Repository in California. Can J Plant Pathol 37:130–135. 10.1080/07060661.2014.999705

