# Supplementary material for: First detection and tracing of grapevine red blotch virus (GRBV) in Australia using tiled amplicon sequencing
Source: Arch Virol. 2025 Jul 10;170(8):177. doi: 10.1007/s00705-025-06366-7 (PMC12245990; doi:10.1007/s00705-025-06366-7)
Supplement: Supplementary file 1 — Supplementary Material 1 [file 705_2025_6366_MOESM1_ESM.docx]

**First detection and tracing of grapevine red blotch virus (GRBV) in Australia using tiled amplicon sequencing.**

Table S1: Varieties sampled for grapevine red blotch (GRBV) virus testing from the WA Germplasm Collection at Manjimup

| **Grapevine variety** | **Grapevine variety** | **Grapevine variety** | **Grapevine variety** |
| --- | --- | --- | --- |
| Alicante Bouschet | Fantasy Seedless | Melon | Saperavi |
| Aligote | Fer | Meunier | Sauvignon Blanc |
| Ansonica | Fernao Pires | Millenium Muscat | Scheurebe |
| Arneis | Fiano | Mondeuse | Schwarzmann |
| Autumn Black | Fiesta | Montepulciano | Sciacarello |
| Barbera | Flame Tokay | Mueller Thurgao | Selma Pete |
| Bastardo | Foex | Muscadelle | Semillon |
| Beauty Seedless | Freedom | Muscat Gordo Blanco WA | Shiraz |
| Biancone | Furmint | Muscat Hamburg | Souzao |
| Borner | Gamay | Muscat Museum | Summer Muscat |
| Brachetto | Gamay de Bouze | Nebbiolo | Sunmuscat |
| Cabernet Franc | Gewürtztraminer | Ohanez | Taminga |
| Cabernet Sauvignon | Gold | Orange Muscat | Tannat |
| Calmeria | Graciano | Paulsen | Tarrango |
| Canada Muscat | Grenache | Perle de Csaba | Teleki |
| Cardinal | Gruner Veltliner | Perlette | Tempranillo |
| Carignan | Harmony | Petit Manseng | Thomuscat |
| Carina | Harslevelu | Petit Verdot | Tinta Amarella |
| Carmenere | Kadarka | Pignoletto | Tinta Cao |
| Carnarvon Muscat | Kerner | Pinot Blanc | Tinta Molle |
| Centennial | Kober | Pinot Gris | Touriga |
| Chambourcin | Kyoho | Pinot Noir | Traminer |
| Chardonnay | Lagrein | Pinotage | Verdehlho |
| Chenin Blanc | Lambrusco | Queen | Millardet |
| Christmas Rose | Lider | Ramsey | Vermentino |
| Cinsaut | LN 33 | Red Globe | Viognier |
| Couderc | Maerlot | Richter | Wortley Hall |
| Crimson Seedless | Magic Seedless | Riesling | Zinfandel |
| Dawn Seedless | Malbec | Rousanne |  |
| Dolcetto | Malvasia Bianca | Ruggeri |  |
| Durif | Mantey | Rupestris du Lot |  |
| Early Madeleine | Maroo Seedless | Sangiovese |  |
| Emperor | Mataro | Santa Paula |  |

Table S2: Varieties sampled for grapevine red blotch (GRBV) virus testing from the Alternative Variety blocks at Wokalup in Western Australia.

| **Grapevine varieties** | **Grapevine varieties** | **Grapevine varieties** | **Grapevine varieties** |
| --- | --- | --- | --- |
| Alicante bouschet | Fer | Montepulciano. | Scheurebe |
| Arneis | Fiano | Pignoletto | Sciacarello |
| Brachetto | Graciano | Pinot Gris | Tannat |
| Carmenere | Harslevelu | Sangiovese | Vermentino |
| Dolcetto | Kadarka | Saperavi |  |
| Durif | Lagrein | Savagnin |  |
